# From training to practice: a longitudinal study on the retention and career entry of young general practitioners in Czechia

**DOI:** 10.1186/s12875-026-03374-7

**Published:** 2026-05-21

**Authors:** Luděk Šídlo, Jan Bělobrádek, Tom Philipp

**Affiliations:** 1 Department of Demography and Geodemography, Faculty of Science, Charles University, Albertov 6, Prague, 12800 Czechia; 2 Department of Preventive Medicine, Faculty of Medicine in Hradec Králové, Charles University, Šimkova 870, Hradec Králové, 500 03 Czechia; 3 Clinic of Rheumatology and Physiotherapy of the Third Faculty of Medicine of Charles University, Thomayer University Hospital, Vídeňská 800, Prague, 14059 Czechia; 4 General Health Insurance Company of the Czech Republic, Board of Directors, Orlická 2020/4, Prague, 13000 Czechia

**Keywords:** General practice, Workforce, Career paths, Young physicians, Czechia

## Abstract

**Background:**

The location of postgraduate training is considered a key determinant for the future practice location of primary care physicians. This study evaluates the effectiveness of the GP training program in Czechia, focusing on the spatial and temporal links between the place of training and subsequent career entry.

**Methods:**

We conducted a longitudinal analysis of contractual data from the General Health Insurance Company (GHIC). While GHIC insures approximately 60% of the Czech population, it maintains contracts with virtually all GP practices providing standard care in Czechia, ensuring near-complete coverage of the primary care provider network. We tracked 661 GP trainees who completed their training between 2009 and 2022. Career paths were analyzed using geolocation mapping at four administrative levels (from municipality to region) alongside data on age, sex, and full-time equivalent (FTE) workloads.

**Results:**

Entry into independent practice is a gradual process: only 55% of doctors established a contract within the first year (44% specifically in GP), increasing to 83% after seven years (79% in GP). A significant gender gap was observed: men entered practice more rapidly and with higher FTEs, while women (representing 73% of the cohort) showed a slower, steadier increase in practice involvement. Geographic retention at the regional level (NUTS 3) reached 65% for part-time and 49% for full-time positions after seven years. Retention at the municipal level (LAU 2) was lower (40% and 28% respectively), often linked to trainees taking over established practices from their trainers.

**Conclusions:**

The study confirms the ongoing feminization of primary care and a growing preference for flexible workloads among young GPs. While the place of training significantly influences future practice location, the transition to independent practice is not immediate. These findings suggest that healthcare workforce planning should account for the specific career trajectories of female physicians and the importance of regional training capacities to ensure long-term stability in primary care.

## Background

Healthcare systems today, regardless of their maturity and financial means, are facing several negative trends, including the uneven distribution of healthcare services to the detriment of rural areas, the ageing of medical professionals, and insufficient recruitment and retention of young doctors in rural regions [1]. This is a global problem, particularly reflected in the European primary care sector [2–3]. For general practitioners (GPs) in Czechia, studies have also demonstrated increasing average ages and an insufficient, regionally uneven turnover of doctors, with the lowest rates observed in rural areas [4].

The motivation of newly trained doctors to work in less attractive, often rural, areas is currently a major research topic [5–10]. While it has long been assumed that the location of training exerts a significant impact on a physician’s future place of practice [11–13], the evidence for this claim remains not entirely convincing [14–18]. A decentralized postgraduate training system has been in place for many years in Czechia; however, at the beginning of the millennium, a sharp decrease in the number of doctors training to become GPs was observed, contrary to expected demographic trends [19].

In 2009, two significant changes were implemented in the organization of postgraduate medical education (i.e., the period from medical school graduation to attaining full qualification for independent GP practice). First, the training was harmonized with EU standards and shortened; the minimum duration was set at three years for medical school graduates, while remaining longer for those retraining from other specialties [20]. The majority of postgraduate training is provided in trainer practices or under the supervision of trainers in specialist outpatient clinics (20 out of a total of 36 months), with the remainder conducted in hospitals [21].

Second, the Ministry of Health of Czechia established a new subsidy program called “residential places” to fund this training [22]. Technically, these subsidies were granted to the training practices that employed the GP trainees, covering most of their salary costs. The distribution of these subsidies across Czechia has been consistently balanced, with the number of “residential places” proportionally corresponding to the size of administrative regions (NUTS3). Participation in the “residential places” program does not impose any time-related or financial obligations on the trainees toward the state or the trainer. Simultaneously, training outside the subsidy system remains possible, typically occurring in the practices of family members of established GPs.

Concurrently, health insurance companies providing public health insurance in Czechia became involved in this educational process. They assumed an administrative role by registering trainees within their contracts with training practices (regardless of whether the training occurred within or outside the subsidy program). Furthermore, they provided financial co-participation by paying bonuses to the training sites. The rationale for this involvement is statutory, as health insurance companies in Czechia are legally responsible for the stability and accessibility of the healthcare provider network [23].

Since 2009, electronic physician registries mapping professional career paths, among other data, have only gradually been emerging in Czechia. Consequently, it is not possible to monitor the outcomes of the changes in the organization of postgraduate GP training using individual-level data from national physician registries. Conversely, contracts between health insurance companies and medical practices were already fully digitalized throughout this entire period.

By analyzing these data, this study aimed to evaluate the outcomes of the new educational program, not only in terms of absolute success (the proportion of trainees entering general practice) but also from a temporal perspective (determining the interval between the completion of training and the start of independent practice). Given the data structure, it was also possible to track the age and sex of trainees, their workloads in general practice as well as in other specialties – as concurrent employment in multiple specialties or for different employers is permitted in Czechia – and the rate of retention within the regions where the physicians underwent their training.

## Data and methods

The data for this study were provided by the General Health Insurance Corporation (GHIC), the largest health insurance company in Czechia. Although GHIC covers approximately 60% of the total population (5.9 million inhabitants), its significance for this research lies in its universal contractual reach. In the Czech healthcare system, virtually all GP practices providing standard primary care are required to maintain contracts with the GHIC to ensure service accessibility for the majority of the population. Consequently, the dataset represents near-complete coverage of the primary care provider network rather than a mere sample of practices. This allowed for an exhaustive mapping of trainee movements across the entire healthcare system.

Regarding primary care, all GP practices providing a standard scope of care maintain contracts with the GHIC. The healthcare system in Czechia is characterized by low patient out-of-pocket costs; patients do not pay for care at the point of service, as the physicians bill the health insurance companies directly. This was a key parameter for our study. Through the payment of financial co-participation (bonuses), GHIC contracts captured trainees in electronic form regardless of the region or their affiliation with the “residential places” subsidy program. Consequently, the data also captured physicians retraining from other specialties – typically older doctors – and not only recent medical school graduates who primarily utilize the subsidy program.

GHIC contractual data from 2009 to 2022 were analyzed [24] on a quarterly basis (using the final month of each quarter, providing four values per year). We identified the final quarters in which physicians were reported as postgraduate trainees at training sites and monitored their subsequent occurrence in the system – specifically, when and where their unique identifiers reappeared in new contracts with the GHIC. To ensure a minimum one-year follow-up period, only those who had completed their training by the fourth quarter of 2021 (2021/4Q) were included in the longitudinal monitoring of their subsequent place of work.

Furthermore, the locations where the physicians underwent training and the practices where they worked following training were subjected to geolocation mapping. This analysis considered four administrative levels: (1) region (NUTS 3), (2) district (LAU 1), (3) MEP (municipalities with extended powers), and (4) municipality (LAU 2). This approach ensured that 99.5% of practices were included in the dataset; the remaining 0.5% were excluded from the analysis due to the impossibility of precisely locating the practice (primarily cases from the beginning of the observation period that could not be revised retrospectively).

The sex and age of the trainees were monitored using a standard five-year interval approach (25–29, 30–34, 35–39, 40+). Their specialization (GP or other) at the new workplace following training was identified, as was their Full-Time Equivalent (FTE) workload for those practicing as GPs (FTE 0, FTE > 0, FTE = 1.0). We clarify that for FTE < 1.0, it was possible to identify the workloads of physicians working at multiple sites across different specializations, including general practice. For the sake of clarity, these findings were not presented separately in the results section, as the study’s primary aim did not include monitoring total cumulative FTEs across different specialties and employers.

In total, 1,001 unique trainees were identified from the dataset, covering 5,891 continuous quarterly intervals at 638 training sites during the observation period (some sites provided training for multiple trainees sequentially). Of this total, 247 trainees were still in training at the end of 2022/4Q, and another 93 did not meet the requirement of at least a one-year interval following the completion of their training. To monitor “trainee movement” within the system, only those who had completed their training by 2021/4Q were included in the analysis, ensuring a minimum one-year follow-up by 2022/4Q. The final sample comprised 661 trainees, of whom 484 were women (73%) and 177 were men (27%). This ratio remained stable throughout the observed period – with no significant decline in either sex during the training process or between individual years – and is consistent with another Czech study investigating the attitudes of GP trainees [25]. Those who completed their training at the age of 25–29 years accounted for nearly one-half of the trainees (45%) in their first year of post-training GP employment; one-third completed training at age 30–34 (31%), 14% were aged 35–39, and 10% were aged 40 or older. Regional coverage corresponded proportionally to the NUTS 3 level in terms of both regional population and the number of participating training sites.

The number of trainees available for analysis naturally decreased as the follow-up duration increased. To maintain data robustness, a minimum threshold of 200 trainees was established for the study group. This resulted in a maximum follow-up period of seven years from the completion of training, a criterion met by a total of 227 trainees (see Table 1).

**Table 1:** Number of trainees by year of completion of training and monitored period since the completion of training

Number of trainees		Monitored period since the completion of training (in years)									
		1	2	3	4	5	6	7	8	9	10
Year of completion of training	2009	9	9	9	9	9	9	9	9	9	9
	2010	16	16	16	16	16	16	16	16	16	16
	2011	36	36	36	36	36	36	36	36	36	36
	2012	68	68	68	68	68	68	68	68	68	68
	2013	39	39	39	39	39	39	39	39	39	
	2014	26	26	26	26	26	26	26	26		
	2015	33	33	33	33	33	33	33			
	2016	32	32	32	32	32	32				
	2017	37	37	37	37	37					
	2018	49	49	49	49						
	2019	78	78	78							
	2020	80	80								
	2021	158									
Total		661	503	423	345	296	259	227	194	168	129

Note: The analys is included trainees with a monitored period since the completion of training of 1-7 years

Source: [24]; author’s own calculations

## Results

**Fig. 1:**
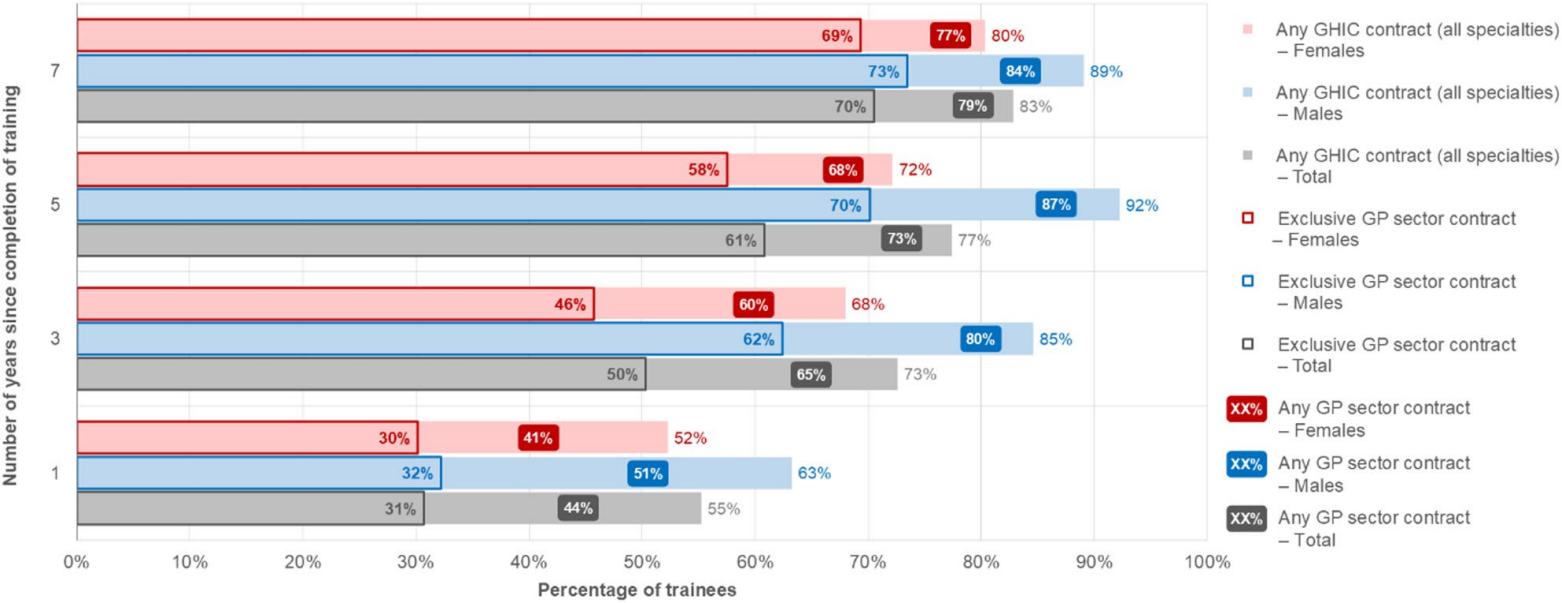
Cumulative entry of trainees into the healthcare system and the GP sector – proportion of the cohort with any GHIC contract, any GP sector activity, and exclusive GP sector. Source: [24]; author’s own calculations

Physicians enter independent practice gradually following the completion of their training (Fig. 1). In the first year post-training, only 55% of newly trained physicians were found to have a contractual relationship with the GHIC, and only 44% worked at least partially in the GP sector (which includes those also practicing in another healthcare specialty). The proportion of physicians working exclusively in the GP sector with an FTE > 0 stood at 31%. Seven years after completing their training, 83% of trainees were in a contractual relationship with the GHIC, and 79% worked at least partially as GPs. The share of those working exclusively in the GP sector with an FTE > 0 reached 70%. Differences by sex were evident; men showed a higher rate of participation as healthcare providers across all monitored years and categories. However, it should be noted that the higher involvement of men in contractual relationships throughout the observation period had a limited impact on the overall results, as men represented only approximately one-quarter of the study cohort.

**Fig. 2:**
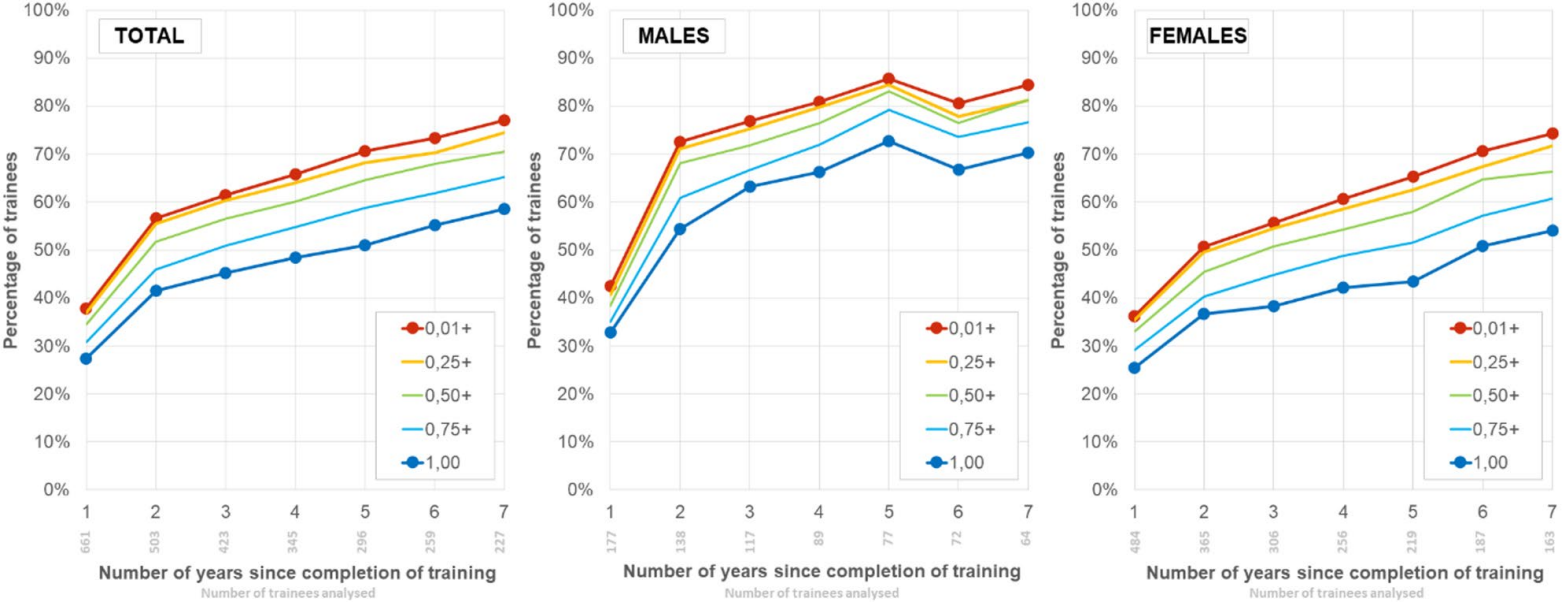
Proportion of trainees who participated in the GP sector after completing training by contracted FTE workload. Source: [24]; author’s own calculations

Differences between the sexes were observed in terms of the FTE workloads in the GP sector following the completion of training (Fig. 2). While the curve for men exhibited a significant increase in the initial years, peaking five years after completing training, the curve for women showed a constant upward trend, which was reflected in the overall results. Furthermore, a significantly lower representation of FTE = 1.0 was observed among female GPs, which may be attributed to general trends in balancing professional and family life.

**Fig. 3:**
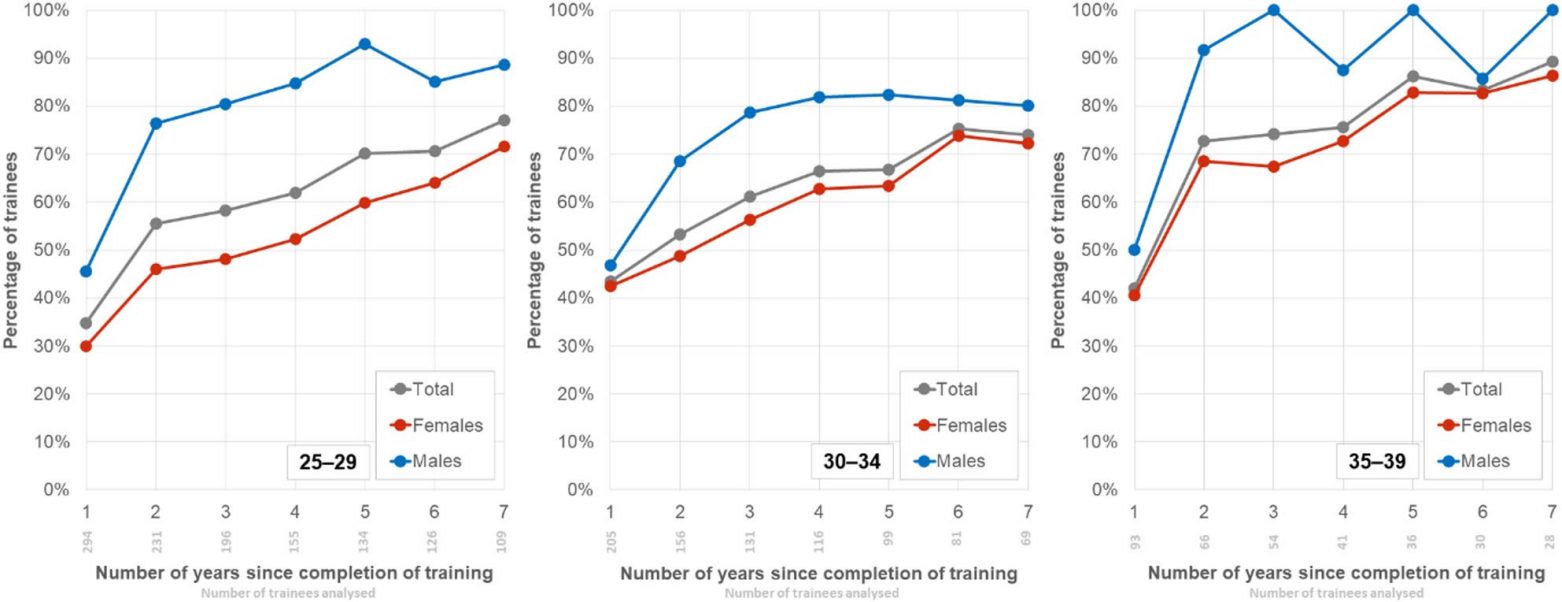
Proportion of trainees who participated in the GP sector after completing training by age category. Note: The age group is stated in terms of the age of the trainee at the end of the training period. Source: [24]; author’s own calculations

The influence of trainee age on the duration of participation in the GP sector following training completion appeared to be minimal (Fig. 3). The curves exhibited similar trajectories across all selected age categories; furthermore, the level of participation in the GP sector was generally higher for men in all age groups. However, since the number of trainees decreased with increasing age (nearly half were in the 25–29 age group and another third in the 30–34 group), the results for the older categories may have been affected by the relatively small sample sizes. Consequently, the 40 + age category was excluded from the graphical comparison to maintain data reliability.

**Fig. 4:**
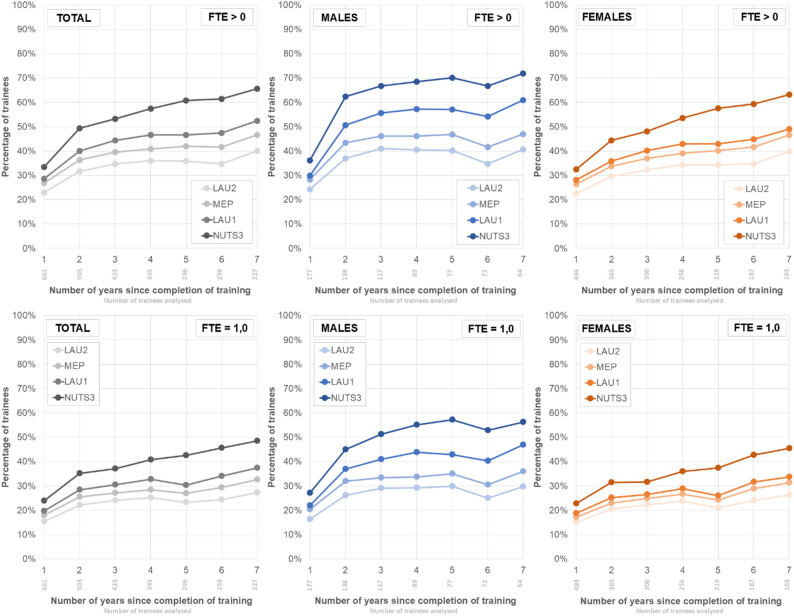
Proportion of trainees who participated in the GP sector after completing training by geolocation level and FTE workload. Source: [24]; author’s own calculations

It was of particular interest to observe the extent to which the regions of training coincided with the regions where the trainees subsequently entered practice (Fig. 4). This spatial retention was analyzed considering two levels of workload: active participation in general practice (GP FTE > 0) and full-time commitment (GP FTE = 1.0). Adherence to the training site location declined as the level of job commitment increased and as the geolocation grid became more granular, with the lowest adherence observed at the municipal level. Furthermore, women exhibited a lower degree of adherence to their place of training than men. Overall, seven years after training completion, the GP FTE > 0 retention rate stood at 40% at the municipal level (LAU 2) and 66% at the regional level (NUTS 3). In comparison, the GP FTE = 1.0 retention rate was 27% at the municipal level (LAU 2) and 48% at the regional level (NUTS 3).

## Discussion

Regarding the complexity and reliability of the data source (GHIC), it is important to emphasize that all standard primary care practices in Czechia are required to maintain contracts with the GHIC. In Czechia, accreditation for postgraduate training is granted only to GHIC-contracted practices, as accreditation is conditional upon specific equipment and personnel requirements, as well as minimum volumes of certain procedures (e.g., patient home visits). Regarding the registration of trainees within health insurance contracts, it can be assumed that the financial incentives (bonuses) ensure high long-term compliance among trainers. It is highly unlikely that accredited training sites would voluntarily forego these financial contributions, particularly since they are legally required to provide trainees with a salary. However, minor inaccuracies during the initial years of the transition to the new training system cannot be ruled out, potentially due to variations in methodological application across individual GHIC regional branches. Such factors may explain the unexpected regional disparities detected in the early data.

International literature provides extensive evidence regarding the importance of the location where physicians receive their training—both undergraduate and postgraduate—in positively shaping the affinity of young doctors toward rural or otherwise underserved areas [26–27]. This factor is considered more influential than the provision of direct financial incentives [28] or restrictive administrative measures, such as practice licenses, mandatory placements, or visa conditions [29]. Nevertheless, research has not yet clearly established the specific determinants that modulate the strength of the bond between the place of training and the future place of practice for newly trained physicians [30–31].

Although a broad spectrum of studies has addressed physician retention in the primary care sector, international comparisons are often hampered by differences in training program structures, intervention measures, and the diverse methodologies applied. A longitudinal study in Norway recorded a decline in the presence of a cohort from the University of Tromsø in rural regions from 60% to 37% between 1979 and 2012 [32]. In Belgium, a 2015 study based on a large sample of students from 1999 to 2013 reported that while 78.5% of graduates worked in primary care, 45.8% practiced on a part-time basis [33]. Similarly, research in Ireland demonstrated a 43% retention rate of GPs in the interval 6–8 years post-graduation [34]. In Portugal, recent changes in incentive schemes led to a 59% increase in retention within underserved areas [35], while less specific outcomes have been reported in recent studies from Germany [36] and Estonia [37]. Research in the USA revealed a 70% retention rate in rural settings over a 20-year period following undergraduate studies [38] and a 47.3% retention rate over a 5-year period following residency programs [39]. In Australia, a 53% retention rate within the respective region was reported following the completion of a regional training program [40]. Our findings regarding the situation in Czechia appear to align with the international trends identified in the literature.

A relatively unexpected finding is the high variability in workloads, particularly in the initial years following training completion. The current educational system in Czechia permits this flexibility due to the absence of mandatory service commitments, even within the “residential places” (rezidenční místa) subsidy program. This allows physicians to balance professional duties with parental roles; however, many also work part-time in general practice while simultaneously practicing in other specialties. A portion of trainees does not practice as GPs at all, apparently considering the specialty as merely one of several future career options. Nevertheless, this variability diminishes as the time since training completion increases.

Regarding geolocation categories, the curves diverge after an initially cohesive first year. This suggests that the first year often involves cases where the trainee takes over the practice directly from the trainer (succession). In contrast, physicians entering independent practice after a longer interval tend to seek opportunities in the broader vicinity of their training site. It should be emphasized that, especially in smaller municipalities, the number of available practices (and thus FTEs) is limited, and not every trainer intends to immediately or automatically hand over their practice to a successor. Consequently, retention at higher regional levels (NUTS 3), rather than at the municipal level (LAU 2), represents a more appropriate criterion for assessing the success of the training system.

While differences in the timing of entry into practice were observed based on sex, the 73% representation of women in the cohort precludes broad generalizations. Men tend to enter practice earlier and at higher rates, but their participation curve flattens after five years. Conversely, for women, we observed a slower but steady increase in contractual relationships across all categories. This is undoubtedly related to the social roles of female physicians, who often establish families at the onset of their independent professional careers. These findings align with international experience, suggesting that female physicians’ approach to work is more relational, encompassing various factors – from partner and family considerations to the selection of colleagues and community relationships [41–42].

### Limitations

The analysis presented in this study has several limitations. Although we utilized the most comprehensive data source available in Czechia at the time of writing, the database inevitably contains minor inaccuracies. Most notably, at the beginning of the observation period, certain verification mechanisms concerning the quality of data transferred from healthcare providers to the GHIC database had not yet been fully implemented. Consequently, in some instances, data lacked precise geolocation of the practice, and minor deviations may have occurred regarding the exact recording of physicians’ FTE workloads. Nevertheless, we believe the data are highly representative and statistically robust; any slight deviations are unlikely to have materially affected the overall conclusions. Furthermore, the validation of such data is currently being enhanced through the National Register of Reimbursed Healthcare Services (NRHZS), managed by the Institute of Health Information and Statistics of the Czech Republic (UZIS), which aggregates data from all Czech health insurance companies. Looking forward, this source will enable even more comprehensive analyses over longer time horizons.

## Conclusions

Our research reveals that after obtaining the qualifications required for independent practice (following the postgraduate certification examination), GPs in Czechia enter the healthcare system gradually. Contracts with the GHIC—a prerequisite for operating an independent practice in the Czech Republic—were held by only 55% of physicians (44% as GPs) one year post-certification, increasing to 83% (79% as GPs) after seven years. This indicates a phased entry not only into the broader healthcare system but specifically into general practice.

Rather than demonstrating a significant influence of physician age on system entry, our research highlights the decisive role of sex. The entry curve for men was relatively steep, peaking after five years, whereas the increase for women was more gradual and consistent. Given the increasing feminization of the GP profession (approximately 75% of new GPs are women), the overall national trend necessarily reflects the female trajectory. Consequently, future healthcare workforce modeling must account for the social roles of female physicians and their career-family life cycle following the completion of training.

Regarding spatial retention, the strongest adherence to the training site was observed in the first year post-certification, likely due to the direct takeover of practices from retiring trainers. Over seven years, the retention rate at the training location reached 40% at the municipal level (LAU 2) and 65% at the regional level (NUTS 3) for physicians with any GP workload (FTE > 0). For those working full-time (FTE = 1.0), the rates were 28% at the municipal level and 49% at the regional level. Women consistently demonstrated lower levels of adherence to their place of training than men.

In terms of qualitative parameters, our findings align with international experience, although direct comparisons remain limited by differences in healthcare systems, educational structures, and varying incentive or commitment schemes. A key contribution of this study is the evidence that physician retention in primary care must be evaluated continuously and over the long term. The seven-year observation period utilized in this study proved sufficient to monitor the diverse trends described. These findings can be further refined as more trainees enter practice, thereby increasing the robustness of the study cohort.

## Data Availability

The data that supports the findings of this study is available from the General Health Insurance Company (GHIC); however, restrictions apply to the availability of this data, which was used for the purposes of this study under contract, and is, therefore, not publicly available. However, the data is available from the authors upon reasonable request and with the consent of the GHIC.

## Abbreviations

FTE: Full Time Equivalent
GHIC: General Health Insurance Company of the Czech Republic
GP: General practitioner
LAU: Local administrative unit
MEP: Municipalities with extended powers
NUTS: Nomenclature of units for territorial statistics
OECD: Organisation for Economic Co-operation and Development
WHO: World Health Organization
